# A Parkinson’s Disease Measurement System Using Laser Lines and a CMOS Image Sensor

**DOI:** 10.3390/s110201461

**Published:** 2011-01-26

**Authors:** Rong-Seng Chang, Jen-Hwey Chiu, Fang-Pey Chen, Jyh-Cheng Chen, Jen-Lin Yang

**Affiliations:** 1 Department of Optics and Photonics, National Central University, 300 Chung-Da Rd., Chung-Li 32001, Taiwan; 2 The Institute of Traditional Medicine, National Yang-Ming University, Taipei, Taiwan; E-Mail: chiujh@mailsrv.ym.edu.tw; 3 Center for Traditional Medicine, Taipei Veterans General Hospital, Taipei, Taiwan; E-Mails: fpchen@vghtpe.gov.tw (F.C.); jlyaug@vghtpe.gov.tw (J.Y.); 4 Department of Biomedical Imaging & Radiological Sciences, National Yang-Ming University, Taipei, Taiwan; E-Mail: jcchen@ym.edu.tw

**Keywords:** Parkinson’s disease, laser triangulation measurement, centroid method, CMOS image sensor, Fast Fourier Transform (FFT)

## Abstract

This paper presents a non-invasive, non-contact system for the measurement of the arterial dorsum manus vibration waveforms of Parkinson disease patients. The laser line method is applied to detect the dorsum manus vibration in rest and postural situations. The proposed measurement system mainly consists of a laser diode and a low cost complementary metal-oxide semiconductor (CMOS) image sensor. Laser line and centroid methods are combined with the Fast Fourier Transform (FFT) in this study. The shape and frequency and relative frequency of the dorsum manus vibration waveforms can be detected rapidly using our Parkinson’s disease measurement system. A laser line near the wrist joint is used as the testing line. The experimental results show an obvious increase in the amplitude and frequency of dorsum manus variation in the measured region in patients suffering from Parkinson’s disease, indicating the obvious effects of the disease. Both in postural and rest state measurements, as the patient disease age increases the vibration frequency increases. The measurement system is well suited for evaluating and pre-diagnosing early stage Parkinson’s disease.

## 1. Introduction

The tremors in patients with Parkinson’s disease have often been studied [1,2]. However, most equipment used for monitoring them is either prohibitively expensive or can interfere with the measurement results due to the need to make physical contact. An optical non-contact technology, based on the optical triangulation principle, is proposed in this study. Laser triangulation is a well-known method for thickness and profile measurements, already used in many industrial fields [3–5]. It has also been used to study the vertical movement of the vocal cords during speech [6]. According to Findley and Gresty [7] a tremor is a “periodic oscillation of a body member”. Different tremors have characteristic local frequencies, and in humans the Parkinson resting tremor generally occurs in the 3–7 Hz frequency range [1–7]. This local frequency is quite different from that of tardive dyskinesia, which tends to occur in the 1–2 Hz frequency range [8,9]. Some of the experiment was using videotaped which cannot get very small (amplitude) vibrations [10]. Here we suggest a laser line triangulation line measurement method which can test not only the very small amplitude (10 μm resolution) vibrations but also the vibration of different local regions simultaneously. In other words we can determine different local frequencies under the same conditions. In this paper, we discuss an example, using our triangulation laser line method to project a laser line onto the dorsum manus. The line crosses three different local vibration regions: the center falls on the hand and the two ends of the local laser line (the reference coordinates) which fall on the table and could be considered as a rigid body.

## 2. Experimental Section

**Figure 1.**
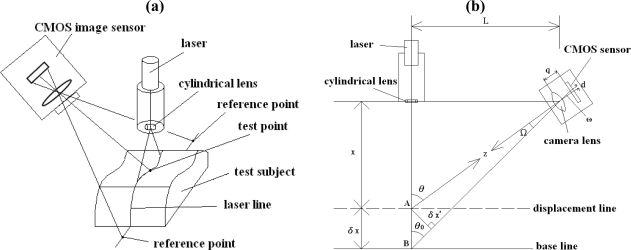
Geometrical layout of the Parkinson’s disease patient arm vibration measurement system [13]. **(a)** The system 3D layout. **(b)** The system 2D layout.

The proposed Parkinson’s disease measurement system combines the Fast Fourier Transform (FFT), the centroid method, and the optical triangulation method [11–13]. The frequency spectrum of the vibration waveform of the dorsum manus of Parkinson’s patient measured at a specified point is obtained by the FFT method. The laser triangulation line method is simple and low cost. It makes it possible to measure the vibration waveforms in a patient’s arm without direct contact. The experimental data show that changes in the vibration waveform of the dorsum manus of Parkinson’s patient can be detected by analyzing the centroid movements of a spot along the projected laser line. The changes at the centroid of the laser spot, which is measured at certain points on the laser line projected onto the patients arm, can be transformed into the variation in vibration frequency. The optical non-contact measurement device consists of a laser diode, a laser driver, and a CMOS image sensor. The geometrical 3D layout of the system is shown in Figure 1(a). The procedure is as follows: (1) The laser diode emits a laser light through a cylindrical lens to form a laser line on the measurement site of the skin surface of the dorsum manus. (2) A laser line image is captured by the CMOS image sensor. (3) Measurements from three chosen points on the line are processed. (4) Through the FFT method, frequencies of the dorsum manus of the test subject are determined.

The geometrical 2D layout of the system is shown in Figure 1(b). X represents the distance between the target object and the collimated lens of the laser diode and δX is the small variation (*i.e.*, the distance between measured points A and B) of the skin surface due to the vibration of the hand. From Figure 1(b), by triangular similarity, the field angle Ω = ω. Therefore, the target distance X is given by:

(1) $$X=\frac{L}{\text{tan}\left(\theta_{0}+{\text{tan}}^{-1}(d/q)\right)}$$

where:

L: The distance between the laser and the CMOS image sensor.
d: The distance between the two spots mapped onto the CMOS image sensor.
q: The focal length of the lens.
Z: The distance between the measured point A and the center C of the lens of the CMOS image sensor.
Ω: The angle between the axis and the measured point A.
δX′: The distance between the measured point A and the optical axis of the lens.
θ0: The angle between the two axes of the CMOS image sensor and the laser.

Differentiating Equation (1) with respect to X, we can get δX, as the result of:

(2) $$\delta X=\frac{Z^{2}d}{\mathrm{qL}}$$

where δX is also regarded as the resolution of the designed Parkinson’s disease measurement system.

**Figure 2.**
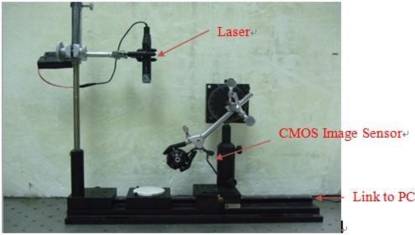
Actual implementation of the proposed system.

The system setup is shown in Figure 2. In our experiments we use a CMOS image sensor (HV7131D, manufactured by Hynix Semiconductor Incorporated) to detect the laser light line which is coming from a 1.3 mW laser diode (Model no. QL63d5sA, MORETEC, Inc.) [13].

The resolution of the system can be increased by introducing a sub-pixel processing technique [11,12]. It should also be noted that about 4–7% power reflection occurs due to differences in the refractive indices of the skin layers for normal incidence of laser light [10]. We use the MATLAB and Origin software packages to develop the signal processing program for calculating the vibration frequency of the dorsum manus as indicated by the laser light line. The signal processing is as follows:

1. Alignment and project the laser line:
  Step1: Project the laser line to the measurement subject.
  Step2: Record the frames for 10 or 20 seconds.
  Step3: Transfer the recorded frames by time divide into image file.
2. Filtering and thresholding: Set the red color filter and intensity threshold to eliminate the light unwanted.
3. Centroid each small region:
  Step1: Select the desired section of the laser line.
  Step2: The laser line block is divided into many small regions.
  Step3: Computing the centroid positions of each region.
  Step4: Synthesis of each centroid position of centroid line’s points.
  Step5: Transform the centroid line’s points position into height.
4. Layout waveform and FFT get the frequency:
  Step1: Draw out the centroid position-time figure.
  Step2: Analyze the trend of centroid line position.
  Step3: Chosen the test point and reference points.
  Step4: Obtain the waveform profile of the test points and reference points.
  Step5: Using the Fast Fourier Transform(FFT) to obtain the output frequency.

**Figure 3.**
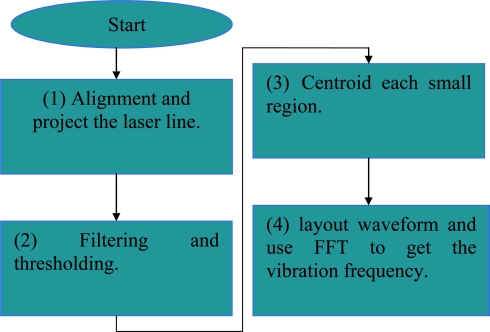
Signal processing flowchart for the proposed Parkinson’s disease measurement system.

The flowchart is shown in Figure 3.

## 3. Calibration and Validation of the Parkinson’s Disease Measurement System

**Figure 4.**
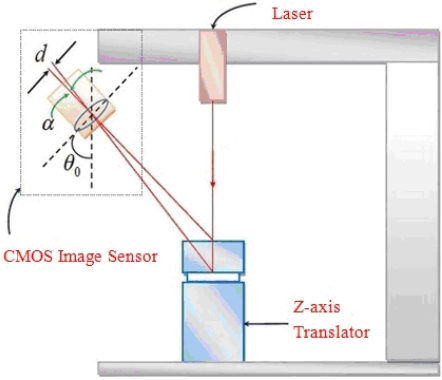
Schematic drawing of the linearity calibration of the Parkinson’s disease measurement system.

**Figure 5.**
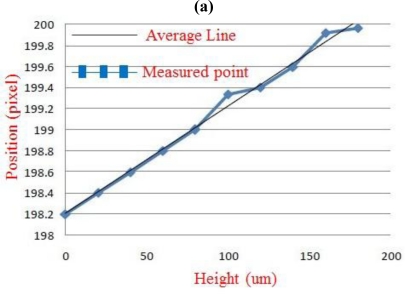

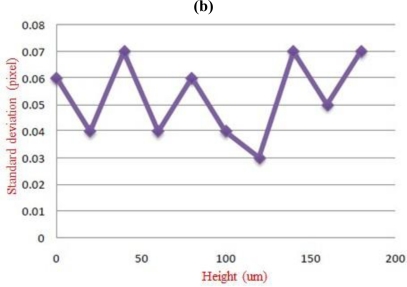
Calibration of the arm vibration amplitude measurement by the proposed Parkinson disease patient measurement system: **(a)** results for the linearity calibration experiments and **(b)** one standard deviation of amplitude calibration.

**Figure 6.**
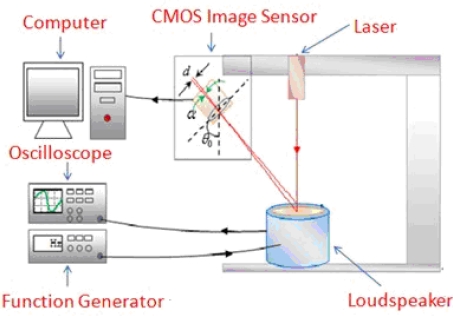
Schematic drawing of frequency calibration for the Parkinson’s disease vibration measurement system.

**Figure 7.**
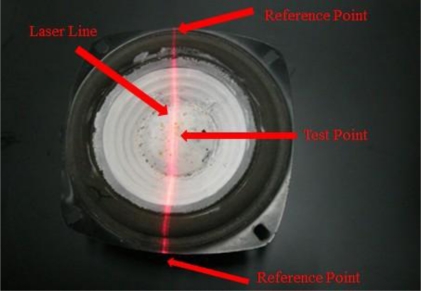
The loudspeaker could be the reference of the frequency calibration of our Parkinson’s disease measurement system.

There are two calibrations in our system: (1) The amplitude measurement calibration, (2) the frequency calibration measurement. In these calibrations, we change the test subject of Figure 1(a) to a standard subject: the precise position translator for amplitude measurement and the precise frequency vibration drum for frequency calibration. The amplitude measurement calibration of the Parkinson’s disease measure system was by a precise translator. A precise translator was used as a support platform to calibrate the linearity of the Parkinson’s disease measure system [13] and is shown in Figure 4. In this experiment, the system measured on the platform the amount of change in z-axis displacement as a correction. The laser line will be drifting up and down. We recorded the centroid position of the laser line by the average distance within 3 seconds. This was done a total of 10 times and each time moved to 0.1 ± 0.01 mm, so the total distance moved is 1 mm in the z-axis direction. The results of the linearity calibration experiments are shown in Figure 5(a). We made the measurements 30 times and get one standard deviation. The results are shown in Figure 5(b). On the other hand, for frequency calibration, we use our laser line to project on a loudspeaker as a test subject which was driven by a function generator (LFC-1300, leaden, Inc), with some specified frequencies. These specified frequencies could be referred as a standard frequency of the drumhead variation, so as to be compared with the laser line variation on it. The layout of the frequency calibration for Parkinson’s disease vibration measurement system is shown in Figure 6. The actual loudspeaker which the projected laser line on it is shown in Figure 7. Here the laser line was projected across the center of the drum center which is the test point, and at the end of the laser line which is on the stable support ring of the variations drum head as the reference points. The reference point is on the rigid unmovable edge part of the drum. When the drumhead vibrated, the variations of the laser line were captured by the CMOS image sensor of the Parkinson’s disease measurement system. The test point vibrations were recorded and transformed to vibration frequency through FFT. The results compared with the function generator’s driven frequencies from 0.5 to 10.0 Hz, are shown in Table 1. In the test, the frame capture rate of the CMOS image sensor of the Parkinson’s disease measurement system was set to 15 frames/sec and the pictures captured by the CMOS image sensor lasted for 10 or 20 seconds for each measurement [13].

## 3. Results and Discussion

Experiments were carried out to demonstrate our non-invasive, non-contact system for measuring Parkinson’s disease patient dorsum manus vibration waveforms of the artery by applying a laser line method to detect vibration. The laser line is projected on the dorsum manus, and the CMOS Image Sensor continuously records the laser line on the hand of the patient for 20 seconds. Through triangulation and the centroid calculation on the surface of the dorsum manus vibration, we can determine involuntary trembling of the dorsum manus in the time domain. The results are then processed by the Fast Fourier transform (FFT) to determine changes in the vibration frequency by appropriately changing the frequency domain. In order to know whether the person has Parkinson’s disease, we focused on patients with the Parkinson’s disease frequency when carrying out the dorsum manus test, using as a control group individuals with a normal general health status for comparing the frequency of the dorsum manus vibration.

**Figure 8.**
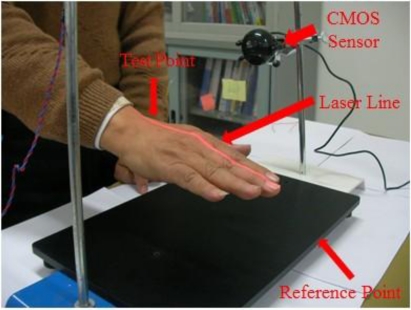
Hand detection method for postural action tremors.

In the experiments, the test subjects were divided into two main groups for comparison and analysis. The first group was comprised of normal healthy individuals not suffering from any major disease while the second was comprised of individuals identified as Parkinson’s disease patients; see Table 2. The first experiment of hand detection method for postural action tremors (hand is floating 15 cm above the testing table) was shown in Figure 8.

**Figure 9.**
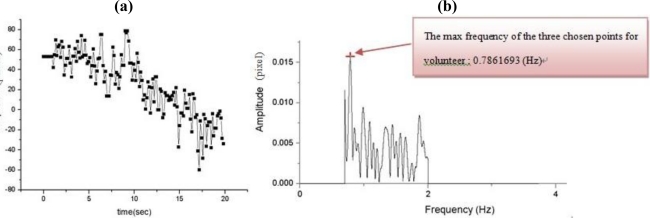
Control group (normal) where the dorsum manus was measured for the postural action tremors by the Parkinson’s disease measurement system: original data for centroid variation of laser line with time and (b) the changes in the vibration frequency of the dorsum manus after FFT (Table 3, Volunteer No.1).

**Figure 10.**
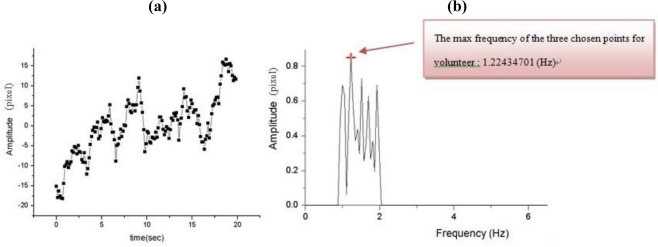
Measurement of the postural action tremor of the dorsum manus of a Parkinson’s disease patient by the Parkinson’s disease measurement system: **(a)** original data showing centroid variation of the laser line over time and **(b)** the changes in the vibration frequency of the dorsum manus after FFT (Table 4, Volunteer No.1).

In Figure 9(a) shows the results of the first experiment for time *vs.* amplitude of the postural action tremor for the control group for dorsum manus vibration as measured by this system. The original data for centroid variation of the laser line with time and the floating state vibration frequency distribution after FFT (maintaining hand posture) are shown in Figure 9(b). Figures 10(a,b) show the same results but for the Parkinson’s disease patient’s dorsum manus.

**Figure 11.**
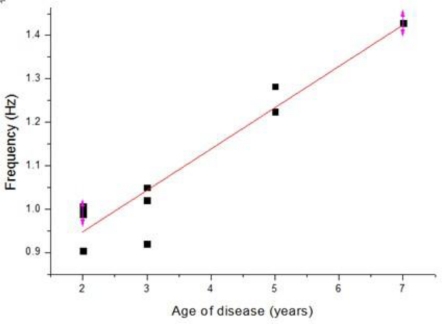
Postural state frequency vibration of the for sum manus in Parkinson’s disease patients dorsum manus.

Some of the measured dorsum manus vibration and frequency results obtained from the different volunteers are shown in Tables 3 and 4, respectively. Table 3 shows the results for the control group, where the normal human dorsum manus frequency ranged from 0.58 to 0.79 (Hz), with an average frequency of 0.75 Hz. Table 4 show the results for people suffering from Parkinson’s disease in the frequency range from 0.90 to 1.43 (Hz), with an average frequency of 1.08 Hz. The average frequency difference Δf posture = Δf_patient_ − Δf normal = 0.33. The P-value of correlation between the age of disease and the vibration frequency is 0.001384 shown the icon in Figure 11 [14]. Since P value less than 0.05, the age of disease and the vibration frequency is very relevant.

**Figure 12.**
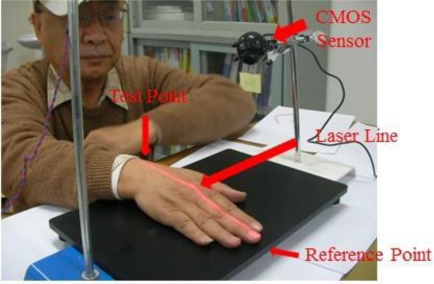
Frequency vibration detection carried out with the hand in a resting state.

**Figure 13.**
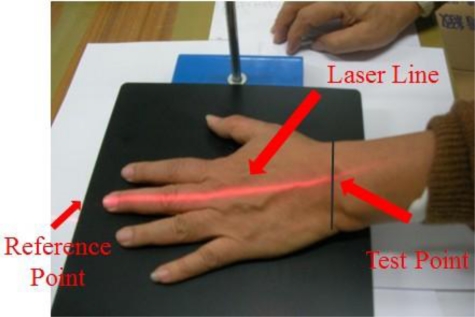
View the laser line at left hand of Parkinson’s disease from the CMOS Image Sensor.

In the next set of experiments, we carried out the detection of rest state (hand is resting on the testing table) frequency vibration. The set-up is shown in Figure 12. In this setup the laser line from the laser diode through a cylindrical lens is projected on the measurement site of the dorsum manus. The image of the laser line on the skin is capture by the CMOS image sensor. The vibration frequency of the two chosen points: one reference point is ahead of the middle finger tip on the table and one test point which is the intersection of the medius line and the last striae transverse of the back of the left hand. Thess test points directly under the laser optical axis are processed by FFT (fast Fourier transform). The vibration frequency of the test point and the reference point measurement system is shown if Figure 12 and the test point position on the back of the left hand is shown in Figure 13.

**Figure 14.**
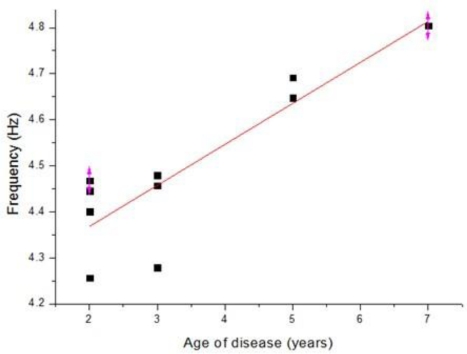
Frequency vibration of the Parkinson’s disease patient’s dorsum manus measured in a resting state after normalization.

The measurement results for the dorsum manus resting tremor frequency obtained from the different volunteers for normal and Parkinson’s disease patients are shown in Tables 5 and 6, respectively. Table 5 shows the results for the control group. The normal human dorsum manus frequency ranges from 0.31 to 0.48 Hz, with an average frequency of 0.41 Hz. Table 6 shows the results for people suffering from Parkinson’s disease. The frequency ranges from 4.25 to 4.8 (Hz), with an average frequency of 4.5 Hz. The average frequency difference is Δf posture = Δf_patient_ − Δf normal = 4.09. From the results in these two tables we can see that the vibration frequency is significantly higher in the Parkinson’s disease patients than in people without the disease. The experimental results are consistent with the references, where in humans, Parkinson resting tremor generally occurs in the 3–7 Hz frequency range. The P-value of correlation between the age of disease and the vibration frequency is 0.009633 shown the graph in Figure 14 [14].

The experimental results show that in the dorsum manus at rest of Parkinson’s disease patients, the average tremor frequency is 4.5 Hz. The dorsum manus postural action tremor frequency of ten patients ranges from 0.5 to 1.5 Hz, with an average frequency of 1 Hz. The dorsum manus resting tremor frequency of the normal control group ranges from 0 to 0.8 Hz, with an average frequency of 0.4 Hz. The postural action tremor frequency ranges from 0 to 1.5 Hz, with an average frequency of 0.75 Hz. The dorsum manus tremor frequency is proportional to the duration of disease. By calculated their difference Δf = f_resting_ − f_postural action_. We get the preliminary data. Δf = 3.5 Hz which is ten times than the control group (Δf = 0.35). In the future, we are going use laser line to detect two different situations simultaneously, for example the frequencies of postural action tremor and the resting tremor by one laser line and get two frequencies dorsum manus simultaneously to cancel the psychology and environment noises.

## 4. Conclusions

With our proposed method we can measure microvibrations of the human hand with a resolution as high as 10 μm. We demonstrate that the application of a CMOS image sensor in a new design for a non-contact, portable, easy-to-use, low cost Parkinson’s disease measurement system is feasible. Both in postural and rest state measurements, as the patient disease age increase the vibration frequency increase. The measurement system is well suited for evaluating and pre-diagnosing early stage Parkinson’s disease.

## Figures and Tables

**Table 1.** Frequency calibration of the Parkinson’s disease measurement system from 0.5 to 10 Hz with a function generator and a loudspeaker.

**Standard Frequency (Hz)**	**Frequency Obtained from the System**	**Error (%)**
0.5	0.512	1.2
0.6	0.585	2.5
0.7	0.703	0.42857
0.8	0.82	2.5
0.9	0.879	2.33
1	0.995	0.5
·	·	·
·	·	·
·	·	·
9.7	9.686	0.14433
9.8	9.725	0.76531
9.9	9.888	0.1212
10	9.935	0.65

**Table 2.** Test subjects were divided into two main groups: **(a)** normal human **(b)** identified as patients of Parkinson’s disease.

**(a)**										

Volunteer No.	1	2	3	4	5	6	7	8	9	10
Age	23	25	24	23	25	24	25	26	25	27
Sex	male	female	male	male	male	male	male	male	male	male

**(b)**										

Volunteer No.	1	2	3	4	5	6	7	8	9	10
Age	53	74	75	79	72	79	78	74	84	51
Sex	male	male	female	male	male	male	male	male	male	male
Age of disease	5	2	5	2	3	3	3	2	2	7

**Table 3.** Dorsum manus vibration frequency of control group (normal human).

**Volunteer No.**	**Control Group Normal Human**
1	0.7861693
2	0.7696269
3	0.7679291
4	0.750475746
5	0.778777985
6	0.79954955
7	0.746645508
8	0.755424003
9	0.749429429
10	0.589022388
Total Average Frequency	0.749304991

* The average frequency of the three chosen points for each volunteer: Hz

**Table 4.** Dorsum manus vibration frequency for Parkinson patients.

**Volunteer No.**	**Parkinson’s Disease Patient**
1	1.22434701
2	0.99113806
3	1.28264925
4	0.9036847
5	1.02028918
6	1.0494403
7	0.91985446
8	1.00571362
9	0.98692481
10	1.42840485
Total Average Frequency	1.081244625

* The average frequency of the three chosen points for each volunteer: Hz

**Table 5.** Dorsum manus resting tremor frequency of the control group (normal human).

**Volunteer No.**	Control Group - Normal Humans*
1	0.42201
2	0.31096
3	0.51086
4	0.44422
5	0.35538
6	0.46643
7	0.37759
8	0.48864
9	0.3998
10	0.33317
Total Average Frequency	0.410906

* The average frequency of the three chosen points for each volunteer: Hz

**Table 6.** Dorsum manus resting tremor frequency of Parkinson’s patients.

Volunteer No	Parkinson’s Disease Patients*
1	4.644733
2	4.46806
3	4.69195
4	4.25651
5	4.4573
6	4.47961
7	4.27882
8	4.44575
9	4.40113
10	4.8043
Total Average Frequency	4.493076

* The average frequency of the three chosen points for each volunteer: Hz
